# Effects of an Early Experience Involving Training in a T-Maze Under either Denial or Receipt of Expected Reward through Maternal Contact

**DOI:** 10.3389/fendo.2013.00178

**Published:** 2013-11-15

**Authors:** Antonios Stamatakis, Anastasia Diamantopoulou, Theofanis Panagiotaropoulos, Androniki Raftogianni, Fotini Stylianopoulou

**Affiliations:** ^1^Biology-Biochemistry Laboratory, School of Health Sciences, National and Kapodistrian University of Athens, Athens, Greece

**Keywords:** neonatal learning, HPA axis, stress response, aggression, serotonergic system, hippocampus, amygdala, prefrontal cortex

## Abstract

The mother is the most salient stimulus for the developing pups and a number of early experience models employ manipulation of the mother-infant interaction. We have developed a new model which in addition to changes in maternal behavior includes a learning component on the part of the pups. More specifically, pups were trained in a T-maze and either received (RER rats) or were denied (DER) the reward of maternal contact, during postnatal days 10–13. Pups of both experimental groups learn the T-maze, but the RER do so more efficiently utilizing a procedural-type of learning and memory with activation of the dorsal basal ganglia. On the other hand, the DER experience leads to activation of the hippocampus, prefrontal cortex, and amygdala in the pups. In adulthood, male DER animals exhibit better mnemonic abilities in the Morris water maze and higher activation of the hippocampus, while they have decreased brain serotonergic activity, exhibit a depressive-like phenotype and proactive aggressive behavior in the resident-intruder test. While male RER animals assume a reactive coping style in this test, and showed increased freezing during both contextual and cued memory recall following fear conditioning.

A large wealth of data from both human and animal studies have documented well that early experiences have long-term effects thus influencing adult brain function and behavior. If the early experiences encompass adversity, they can lead later in life to psychopathology, particularly maladaptive stress responses, anxiety, and depression. In the animal models employed to study the effects of early experiences the major parameter modified is the care offspring receive, through altered maternal behavior (1–5). In our laboratory we have developed a novel experimental model of early experiences, which, in addition to altering maternal behavior, also involves a component of neonatal learning by the pups (6). More specifically, during postnatal days 10–13 pups are exposed to a T-maze, one arm of which leads to the mother-containing cage; one group of pups receives the expected reward (RER) of maternal contact by being allowed to be retrieved by the mother upon reaching her cage, while the other group is denied this reward (DER) by blocking access to the mother-containing cage. Interestingly, both groups of pups learn the T-maze, but – as expected – the RER do so more efficiently, by employing a procedural type of learning with activation of the dorsal basal ganglia, as shown by c-Fos immunoreactivity (6). On the other hand the DER experience leads to activation of the hippocampus and the prefrontal cortex, indicating increased vigilance and appraisal of environmental cues. Furthermore, the DER experience resulted in activation of the amygdala on the first day of training (postnatal day 10, PND10) and on that same day an increase in corticosterone was observed (7), which could mediate the early activation of the amygdala since it has been shown that circulating corticosterone controls the maturation of this brain area (8). The increase in corticosterone indicates that exposure to the DER experience for the first time is stressful for the pups and the stress-inducing factor is probably the denial of maternal contact. These results complement those of Sullivan et al. who established that the presence of the mother during early postnatal development acts to inhibit stress-induced corticosterone increase (9).

It should be noted that pups of both groups are returned to the home cage containing their mother and littermates immediately after the end of the exposure to the T-maze procedure: approximately 10 min. When back in the home cage, both RER and DER pups receive increased maternal care compared to the control (non-handled) pups (7), resembling in this respect animals subjected to the neonatal handling paradigm (1, 4, 5). Our model of early experience also bears resemblance to that of Sullivan et al. in that in both there is learning on the part of the pups, and the force motivating it is the mother; in our model the pups learn the position of the mother within the T-maze, while in that of Sullivan et al. the odor associated with the mother (10, 11).

One of the focuses of our studies was the effect of these two early experiences, one with a positive emotional valence (the RER), and the other of minor adversity (given that pups receive increased maternal care soon after exposure to the T-maze – the DER) on adult hypothalamic-pituitary-adrenal (HPA) axis function, since it is well known that it is a primary target of early experiences (12). Neither the RER nor the DER experience affected basal plasma corticosterone levels (13). However, the stress-induced corticosterone response of each group differed, depending on the characteristics of the stressful stimulus: when it was highly aversive within the natural behavioral repertoire of the animals, such as after exposure to an aggressive resident male in the resident-intruder test, there were no differences among the three groups (RER, DER, and Control) and all animals increased their corticosterone levels dramatically (14). Following a 15 min forced swim test, which is fairly aversive, but rodents are exposed to it only within the framework of laboratory experiments, all animals increased their plasma corticosterone, but the DER attained higher levels than either the RER or the controls, which did not differ between them (13). However the stress-induced corticosterone response of the DER animals cannot be characterized as “pathological,” since it was contained, reaching basal levels by 2 h, just like that of the other two groups. Interestingly, when animals were exposed to a stressful stimulus of a very short duration (1 s electric shock of 0.5 mA) within the setting of a fear conditioning paradigm, both RER and DER animals attained lower corticosterone levels than the controls (7), indicating that both early experiences programed the HPA axis to give a blunted response to temporally transient stressful stimuli, a characteristic which could be of adaptive value.

Notably, other components of the HPA axis, such as glucocorticoid receptors (GRs) in the hippocampus, corticotrophin-releasing hormone (CRH) levels in the paraventricular hypothalamic nucleus (PVN) and amygdala, and type 1 CRH receptors (CRH-R1) in the hippocampus and the amygdala were affected only by the DER experience (13).

More specifically GR levels in the hippocampus were higher in the DER animals than either the control or the RER (whose GRs were at the control levels), in spite of the fact that both DER and RER animals received the same, increased maternal care. This finding indicates that the elevated hippocampal GR levels induced by increased maternal care, documented using other early experience models (neonatal handling, high licking and grooming mothers) (15, 16), can be modulated by other factors possibly related to the pups’ behavior.

Another key player in the stress response is the CRH system (CRH and its type 1 receptor). The DER animals had lower CRH-R1 levels in the hippocampus and the amygdala, areas known to participate in the control of stress-induced CRH release (17). On the other hand, although the DER experience did not affect basal CRH levels in the PVN or amygdala, following the stressful stimulus of forced swimming CRH levels remained high 2 h after the termination of the stress in the DER animals (13). The sustained CRH expression in the DER animals following stress could be a reflection of a less efficient feedback control circuit of CRH release, mediated by the lower CRH-R1 levels in brain areas, such as the hippocampus and amygdala, participating in this circuit.

Animals receiving increased maternal care as neonates have improved cognitive abilities as adults, as shown in studies using neonatal handling or the high licking and grooming mothers (2, 18–21). Based on this, we assessed the abilities for learning and memory of the adult male animals subjected as neonates to the RER or DER experience. We employed two different tests: the Morris water maze (MWM), which measures spatial reference learning and memory and is hippocampus dependent, and fear conditioning, which has a strong emotional component and is dependent on both the amygdala and the hippocampus. Interestingly, the DER animals had better mnemonic abilities in the MWM test (22), while the RER in the fear conditioning (7). The improved memory of the DER animals was accompanied by increased levels of the transcription factor pCREB in the hippocampus, indicating increased activation and plasticity related processes in this brain area. It should be remembered that the DER experience resulted in increased activation of the hippocampus in the neonatal period (6). It is thus possible that this early activation of the hippocampus programed its function rendering it more efficient in adulthood. On the behavioral level the DER experience involves an element of “delayed” reward, since the pups are being denied maternal contact during the T-maze training, but do receive it soon after, upon return to the nest. This experience could prepare them to deal efficiently with situations in adulthood when an expected reward is not delivered, such as in the MWM memory trial, where the platform (learned “reward”) is missing. On the other hand the RER neonatal experience, which lacks elements of adversity, renders the animals as adults more prone to remember emotionally negative events such as those eliciting fear. The above described results of the early experiences of our model are in agreement with the match-mismatch hypothesis, which states that if two events during critical periods of life “match” in being mildly, and not severely, stressful, their interaction can be beneficial resulting in a more adaptive phenotype (23).

The serotonergic system of the brain is strongly affected by early experiences. The DER neonatal experience of our model resulted in decreased serotonergic activity as assessed by the lower levels of serotonin in the prefrontal cortex and amygdala, of 5-HT1A receptors in the hippocampus, and of increased serotonin transporter in the amygdala of the adult brain (14).

Serotonergic neurotransmission is intimately involved in the etiopathogenesis of depression and the control of aggression. Interestingly, the adult DER animals were more vulnerable to express depressive-like behavior as assessed by increased immobility time in the forced swim test (13). It is worth mentioning that immobility time has been considered an expression of a passive coping strategy, which could be “adaptive” in the face of an inescapable stressful situation. Even in humans, depression has been viewed as an adaptive response developed through evolution to enable efficient management of complex challenging situations (24). The DER neonatal experience also affected aggressive behavior in both adolescence and adulthood. During adolescence the DER animals displayed play behavior with strong aggressive characteristics (14), similarly to children that have been maltreated or neglected (25). Relevantly, adolescent aggressive-like play is considered as a lack of abilities to integrate into peer groups and could be a prognostic factor for inappropriate social behaviors in adulthood. Moreover, as adults the DER male animals showed more proactive behaviors in the resident-intruder test, indicating a maladaptive response in the face of defeat (14). It has been shown by others that the proactive coping style is associated with increased offensive aggression, impulsivity, and behavioral inflexibility (26, 27), and it is the impulsive form of aggression which is increased by early life adversity (28, 29). Studies in humans have shown that early childhood trauma is often associated with enhanced aggression, which correlates with increased suicide attempts (30). Furthermore, clinically a strong relationship between depression and aggression is generally observed (31, 32).

A wealth of data has been accumulating lately that the consequences of early experiences are effected through epigenetic changes, which include DNA methylation and histone modifications, resulting in altered expression in relevant genes (33–35). Within this framework, it has been shown that the DER experience results in higher levels of phospho(Ser10)-acetyl(Lys14)-Histone-3 in the amygdala of the adult animals (7), while preliminary work has revealed differences in DNA methylation induced by the RER and DER experience throughout areas of the limbic system. It is thus evident that this line of research is highly promising as to the possibility of unraveling the molecular mechanisms underlying the behavioral and neurochemical effects of the early experiences of our model.

The characteristics of the DER neonatal experience, which involves a short separation from the mother and denial of contact with her, in spite of her presence, are reminiscent of human situations of maternal neglect. Furthermore, as presented throughout the text (see Table 1), the behavioral phenotype of the DER animals bears a strong resemblance with that of human adolescents and adults which have been neglected as children. Thus, the DER neonatal experience could provide a good animal model in which to study the long-term effects of early life experiences of mild adversity, which is important, since they are more frequent than those of severe negative valence, such as child abandonment or physical or sexual abuse.

**Table 1:** **Effects of the DER and RER neonatal experience of our model in male rats – compared to controls**.

DER	RER
**NEONATAL LIFE (PND10–13)**	
Activation of hippocampus, prefrontal cortex, and amygdala	Activation of basal ganglia
Increased vigilance and appraisal of environmental cues	Procedural type of T-maze learning
Increased plasma corticosterone on PND10	
Receive increased maternal care	Receive increased maternal care
**ADOLESCENCE**	
Play behavior with strong aggressive characteristics	
**ADULT LIFE**	
No effect on basal plasma corticosterone levels	No effect on basal plasma corticosterone levels
Higher, but temporally restrained, forced swim stress-induced increase in corticosterone levels	
Lower foot shock-induced increase in corticosterone levels	Lower foot shock-induced increase in corticosterone levels
Higher GR levels in the hippocampus	
Lower CRH-R1 levels in the hippocampus and the amygdala	
Sustained forced swim stress-induced increase in PVN and amygdalar CRH levels	
Better mnemonic abilities in the MWM test	Enhanced fear memory
Increased levels of pCREB in the hippocampus following MWM training	
Lower levels of serotonin in the prefrontal cortex and amygdala	
Lower levels of 5-HT1A receptors in the hippocampus	
Increased levels of serotonin transporter in the amygdala	
Increased immobility time in the forced swim test	
More proactive behaviors in the resident-intruder test	

## Conflict of Interest Statement

The authors declare that the research was conducted in the absence of any commercial or financial relationships that could be construed as a potential conflict of interest.

## Abbreviations

CRH, corticotrophin-releasing hormone; CRH-R1, type 1 CRH receptor; DER, denied expected reward; GR, glucocorticoid receptor; HPA, hypothalamic-pituitary-adrenal; MWM, Morris water maze; PND, postnatal day; PVN, paraventricular hypothalamic nucleus; RER, received expected reward.
